# Human Milk Oligosaccharides: Shaping the Anti-Infective Status in Infancy

**DOI:** 10.3390/microorganisms14061261

**Published:** 2026-06-03

**Authors:** Oana-Raluca Temneanu, Otilia Novac, Adriana Mihai, Felicia Trofin, Otilia Elena Frăsinariu, Paula Popovici, Roxana Șerban, Alice Nicoleta Grudnicki, Ileana Katerina Ioniuc, Carmen Liliana Barbacariu, Bianca Simionescu

**Affiliations:** 1 Grigore T. Popa University of Medicine and Pharmacy, 700115 Iași, Romania; temneanu.oana@umfiasi.ro (O.-R.T.); otilia.novac@umfiasi.ro (O.N.); felicia.trofin@umfiasi.ro (F.T.); frasinariu.otilia@umfiasi.ro (O.E.F.); paula.popovici@umfiasi.ro (P.P.); roxana-n-serban@umfiasi.ro (R.Ș.); alice.azoicai@umfiasi.ro (A.N.G.); ileana.ioniuc@umfiasi.ro (I.K.I.); carmen.barbacariu@umfiasi.ro (C.L.B.); 2 “Sf. Maria” Children Emergency Hospital, 700309 Iași, Romania; 3 Sf. Spiridon County Clinical Emergency Hospital Iași, 700111 Iași, Romania; 4 Department of Mother and Child Medicine, Faculty of Medicine, Iuliu Hațieganu University of Medicine and Pharmacy, 400012 Cluj-Napoca, Romania; bianca.simionescu@umfcluj.ro; 5 Children Emergency Hospital, 400012 Cluj-Napoca, Romania

**Keywords:** human milk oligosaccharides, anti-infective, immunity, infant microbiome, infant formula

## Abstract

Human milk is widely recognised as the optimal source of nutrition for newborns and infants, providing not only an ideal macronutrient composition but also a range of bioactive components that exert important non-nutritional functions, and as such it represents the first functional food consumed in early life. Among these bioactive components, the human milk oligosaccharides (HMOs)—a structurally diverse family of glycans present in human milk at concentrations 100- to 1000-fold higher than in the milk of other mammalian species—have emerged as multifunctional contributors to the establishment of the intestinal microbiome, immune development, anti-infective defence, and epithelial barrier integrity during a developmental window characterised by immune immaturity. The aim of the present narrative review is to synthesise current evidence on the anti-infective properties of HMOs in infancy and to integrate, within a single framework, five interconnected mechanisms through which HMOs protect the infant against infection: glycan-mimicry-based competitive inhibition of pathogen adhesion, direct antimicrobial and antibiofilm activity, selective prebiotic shaping of the gut microbiome, modulation of innate and adaptive immune responses, and reinforcement of mucosal barrier integrity in the gut and lungs. Breastfeeding constitutes a natural strategy for anti-infective protection in early childhood, while infant formulas supplemented with biotechnologically produced HMOs that are structurally identical to those in human milk provide measurable benefits for non-breastfed infants.

## 1. Introduction

What makes human milk the gold standard for infant nutrition is not merely its macronutrient content but also its extraordinary biological complexity. A breastfed newborn receives, alongside proteins, fats, and lactose, a remarkable array of bioactive components whose physiological effects have kept researchers occupied for over a century. Foremost among these are the human milk oligosaccharides (HMOs): structurally diverse, biologically active glycans present in concentrations that considerably exceed those found in the milk of any other mammalian species [1,2].

The World Health Organization recommends exclusive breastfeeding for the first six months of life, with continued breastfeeding alongside complementary foods for up to two years or beyond. In parallel, major paediatric societies, including the European Society for Paediatric Gastroenterology, Hepatology and Nutrition (ESPGHAN), support exclusive breastfeeding for approximately six months and continuation thereafter for as long as mutually desired [3,4]. These recommendations rest on robust evidence linking breastfeeding with reduced infant morbidity and mortality, improved immune development, and long-term metabolic and cognitive advantages.

The immunological significance of human milk is greatest precisely during this early window: the first six months of life.

Understanding which components of human milk confer these benefits and how they operate has direct implications for the millions of infants who cannot be exclusively breastfed.

By weight, human milk oligosaccharides (HMOs) are the third most abundant solid component of human milk, present at concentrations of 20–25 g/L in colostrum and declining to approximately 10–15 g/L in mature milk [5,6]. In comparison, bovine milk contains oligosaccharide concentrations that are 100- to 1000-fold lower [7]. This marked quantitative disparity suggests an important functional role.

While the prebiotic and immune-modulatory functions of HMOs have received substantial attention, a body of evidence has emerged over the past decade demonstrating that HMOs also function as direct antimicrobial and antibiofilm agents [8,9]. This dimension of HMO biology’s capacity to inhibit pathogen adhesion, disrupt biofilm architecture, potentiate antibiotic efficacy, and modulate mucosal immune homeostasis positions HMOs not merely as nutritional supplements but as a comprehensive, multifunctional anti-infective system evolved to protect the vulnerable infant during a critical window of immune immaturity.

The present review focuses specifically on the anti-infective properties of HMOs in infancy. We examine five interconnected mechanisms through which HMOs shape the infant’s resistance to infection: competitive inhibition of pathogen adhesion via glycan mimicry; direct antimicrobial and antibiofilm activity against clinically relevant pathogens; selective prebiotic modulation of the gut microbiome to establish colonisation resistance; modulation of innate and adaptive immune responses; and reinforcement of epithelial barrier integrity in both the gut and lungs. We further discuss the clinical translation of these findings, including the integration of HMOs into infant formula, the emerging synbiotic approach combining HMOs with specific probiotic strains, and future therapeutic directions.

## 2. Materials and Methods: Literature Search Strategy and Selection Criteria

**Figure 1:**
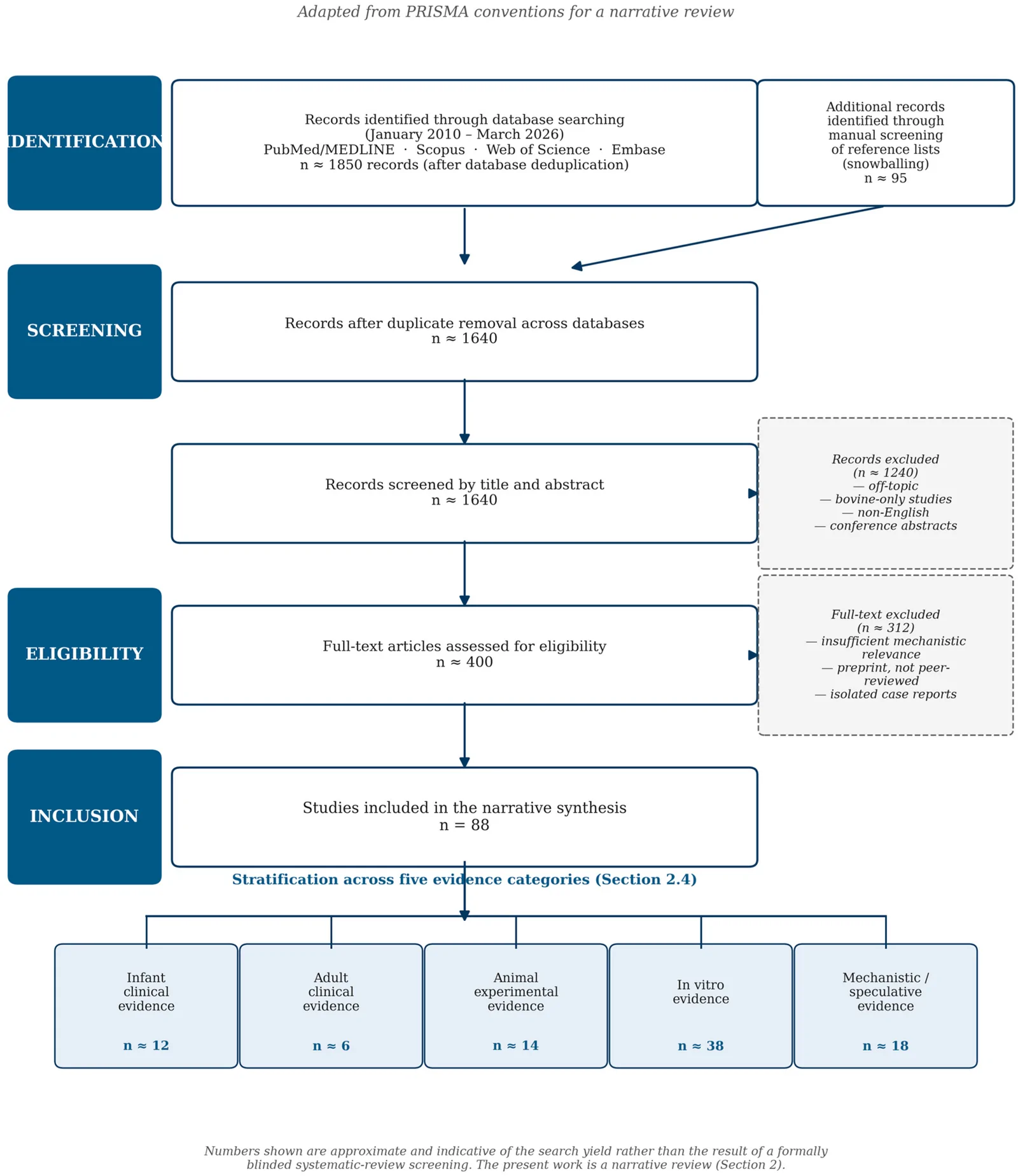
Flow diagram of the literature search and study-selection strategy adopted for the present narrative review on the anti-infective properties of human milk oligosaccharides. The four-stage process—identification, screening, eligibility, and inclusion—follows the structural conventions of PRISMA, adapted to the narrative-review format. Records were retrieved from PubMed/MEDLINE, Scopus, Web of Science, and Embase (January 2010–March 2026), supplemented by manual screening of the reference lists of included studies. The 88 references retained in the final manuscript are stratified across the five evidence categories detailed in Section 2.4.

The present work is a narrative review and was therefore not designed as a systematic review or meta-analysis; nonetheless, the literature search and the selection of cited studies were conducted according to a structured, reproducible protocol, the principal elements of which are summarised below and depicted graphically in Figure 1.

### 2.1. Databases and Search Period

Four electronic databases were interrogated: PubMed/MEDLINE, Scopus, Web of Science Core Collection, and Embase. The searches covered the period from January 2010 to March 2026 inclusive, with explicit prioritisation of publications from the last five years (2021–2026) in order to capture the most recent mechanistic and clinical advances in the field. A small number of seminal works published before 2010 were retained on the basis of their foundational role in establishing the central concepts of human milk oligosaccharide biology, in particular the original characterisation of fucosylated and sialylated species and the first prospective clinical evidence of HMO-mediated protection against infant diarrhoea; this decision is documented transparently below as a methodological consideration. The database searches were complemented by manual screening of the reference lists of all included reviews and primary studies (snowballing), in order to identify additional relevant publications not captured by the structured queries.

### 2.2. Search Terms and Boolean Strategy

The principal search string combined two thematic blocks via the Boolean operator AND. The first block defined the molecular target and included the terms (“human milk oligosaccharide” OR “HMO” OR “HMOs” OR “2′-fucosyllactose” OR “2′-FL” OR “3-fucosyllactose” OR “lacto-N-tetraose” OR “lacto-N-neotetraose” OR “sialyllactose” OR “3′-sialyllactose” OR “6′-sialyllactose” OR “disialyllacto-N-tetraose” OR “DSLNT”). The second block defined the biological domain and combined the terms (“anti-infective” OR “antimicrobial” OR “antibiofilm” OR “antiviral” OR “prebiotic” OR “microbiome” OR “immunomodulation” OR “immune” OR “epithelial barrier” OR “tight junction” OR “respiratory syncytial virus” OR “influenza” OR “rotavirus” OR “norovirus” OR “necrotising enterocolitis” OR “necrotizing enterocolitis” OR “NEC” OR “infant” OR “neonate” OR “preterm”). The two blocks were joined by AND, and the search was restricted to publications written in English by means of the relevant database filters. Where supported by the database, MeSH terms were used in addition to free-text searches.

### 2.3. Inclusion and Exclusion Criteria

Eligible records included peer-reviewed original research articles, systematic and narrative reviews, meta-analyses, randomised controlled trials, prospective and retrospective cohort studies, mechanistic *in vitro* studies, and preclinical animal experiments addressing the composition, biosynthesis, anti-infective mechanisms, immunological effects, or clinical applications of human milk oligosaccharides. Authoritative regulatory and institutional documents (EFSA Scientific Opinions, US FDA GRAS notifications, World Health Organization recommendations, and European Commission Implementing Regulations) were also included where directly relevant. Records were excluded if they were isolated case reports without mechanistic or epidemiological relevance, conference abstracts without subsequent peer-reviewed publication, non-peer-reviewed preprints, or studies focused exclusively on bovine or non-human primate milk oligosaccharides without explicit comparison or extrapolation to human biology.

### 2.4. Stratification of Evidence by Study Type

Evidence retrieved through the search procedure described above was not pooled across study types but was instead retained, presented, and discussed in clearly distinguishable categories. Five tiers of evidence were maintained throughout the manuscript and are explicitly labelled in each summary table: (i) infant clinical evidence, comprising randomised controlled trials and prospective cohort studies in term and preterm neonates and infants; (ii) adult clinical evidence, comprising trials in healthy adult volunteers used principally to characterise pharmacokinetics, microbiome modulation, and synbiotic engraftment; (iii) animal experimental evidence, comprising studies performed in murine, piglet, chicken, and other vertebrate models; (iv) *in vitro* evidence, comprising experiments in cell lines, primary cells, organoids, intestinal enteroids, and binding/biochemical assays; and (v) mechanistic or speculative evidence, comprising structural-biology, computational, and hypothesis-generating studies that propose mechanisms not yet validated experimentally. Throughout the manuscript, the type of evidence supporting each statement is made explicit, and the summary tables include a dedicated “Evidence type” column to facilitate the reader’s appraisal of the strength and translational relevance of each finding.

### 2.5. Selection of Studies for Narrative Discussion

Because narrative reviews do not lend themselves to formal quality scoring, prioritisation of studies for in-depth discussion was guided by four explicit criteria, applied jointly. The first criterion was mechanistic relevance to the five anti-infective domains around which the present review is structured (anti-adhesion, antimicrobial and antibiofilm activity, prebiotic shaping of the microbiome, immune modulation, and epithelial barrier reinforcement). The second criterion was strength and reproducibility of the experimental evidence, with preference accorded to randomised controlled trials over observational studies, to studies including replication or independent validation, and to mechanistic studies supported by structural or biochemical confirmation. The third criterion was recency and citation profile within recent authoritative reviews, used to identify studies that have shaped the current consensus in the field. The fourth criterion was structural and clinical complementarity, that is, the inclusion of studies that fill specific gaps in the mechanistic narrative or that bring together previously disjointed lines of evidence. Studies that did not satisfy any of these criteria were either omitted or cited only briefly for context. The final reference list comprises 88 entries, of which approximately 80% were published within the last five years.

### 2.6. Methodological Limitations

Three principal limitations of the present search strategy should be acknowledged. First, restriction of the literature search to publications in English may have led to the omission of relevant work published in other languages, although the field of HMO research is overwhelmingly disseminated in English-language journals. Second, despite the explicit prioritisation of recent studies, several seminal references published before 2010 have been retained where these constitute primary, irreplaceable sources for the foundational concepts of HMO biology—in particular [2] for the original characterisation of HMOs as multifunctional bioactive glycans, ref. [5] for the consolidated functional biology framework, ref. [10] for the demonstration of glycan-mediated anti-adhesion against *Campylobacter jejuni*, and ref. [11] for the first prospective clinical evidence of HMO-mediated protection against infant diarrhoea; this decision was taken in order to preserve historical attribution and to avoid the introduction of citation drift through reliance on secondary sources. Third, although the prioritisation criteria described in Section 2.5 were applied consistently, the present work remains a narrative rather than a systematic review, and the selection of studies for in-depth discussion is unavoidably influenced by the authors’ mechanistic framework. Readers seeking a fully systematic and quantitative synthesis of any individual sub-domain (for instance, randomised trials of HMO-supplemented infant formulas in respiratory infection) are referred to the dedicated systematic reviews and meta-analyses cited at the relevant points in the manuscript.

## 3. Structure, Composition, and Variability of HMOs

Over 200 structurally distinct HMOs have been catalogued to date, built from five monosaccharide units: glucose, galactose, N-acetylglucosamine, fucose, and the sialic acid N-acetylneuraminic acid [5,12]. Every HMO contains a lactose core that can be extended by the stepwise addition of lacto-N-biose or N-acetyllactosamine units and then decorated at the non-reducing end with fucose residues, sialic acid residues, or both [5,13].

**Figure 2:**
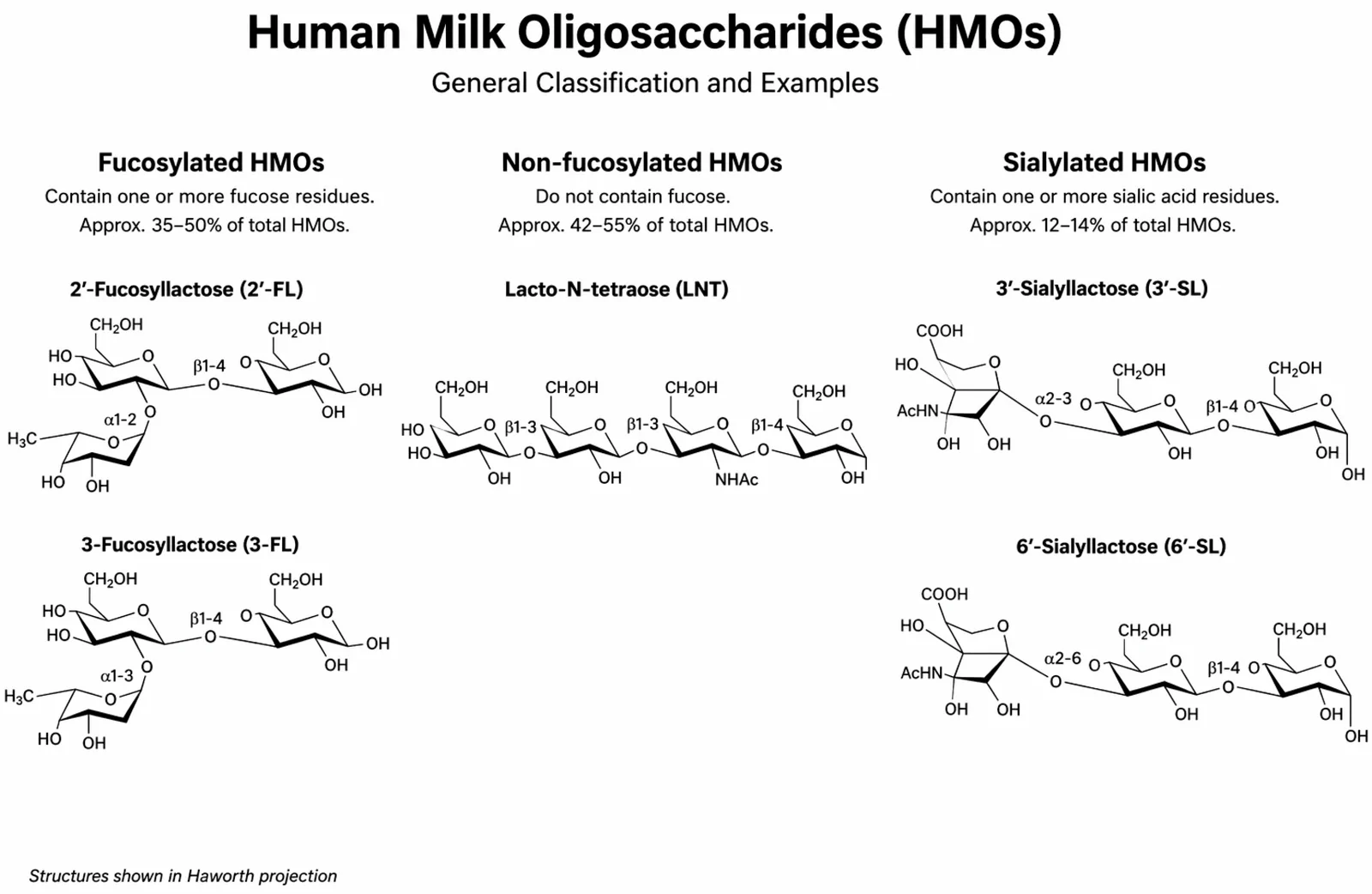
Structural classification of human milk oligosaccharides into fucosylated, non-fucosylated, and sialylated species, with representative examples shown in Haworth projection. Author’s original figure based on data from reference [2].

Three broad structural categories of HMOs are recognised. Neutral fucosylated species, which include the highly abundant 2′-fucosyllactose (2′-FL), account for 35–50% of total HMOs. Neutral non-fucosylated species, among them lacto-N-tetraose (LNT) and lacto-N-neotetraose (LNnT), represent 42–55%. Acidic sialylated HMOs, including 3′-sialyllactose (3′-SL) and 6′-sialyllactose (6′-SL), make up the remaining 12–14% [14]. Neutral species collectively exceed 75% of the total HMO pool, and 2′-FL alone may constitute nearly 30% of all HMOs in women who express a functional FUT2 enzyme (secretor phenotype) [2]. The structural classification of HMOs into these three categories, with representative examples shown in Haworth projection, is illustrated in Figure 2.

HMO composition is dynamic. It varies with gestational age, lactation stage, maternal secretor and Lewis blood group status, geographic and ethnic background, and maternal nutritional status [12,15]. The maternal phenotype results from genetic polymorphisms in the Secretor (Se) and Lewis (Le) genes, encoding fucosyltransferase FUT2 and FUT3, respectively. Four phenotypic variants can be distinguished, each producing a characteristic HMO profile [16,17].

Crucially, however, this inter-individual variability does not imply functional deficiency: regardless of secretor status or Lewis blood group phenotype, all lactating women synthesise the core repertoire of human milk oligosaccharides, ensuring that the fundamental functions of HMOs are universally conferred upon the breastfed infant.

HMOs are notably resilient: they withstand pasteurisation, lyophilisation, and both cold and heat storage without significant structural degradation [18]. The relationship between milk composition and infant microbiota was recently explored in a pilot study by Fricker et al., demonstrating that specific HMOs correlate with specific commensal taxa and their functional glycoside hydrolase gene repertoires [19].

## 4. Individual HMOs’ Anti-Infective Properties

While the subsequent sections of this review examine the anti-infective mechanisms of HMOs, thematically addressing anti-adhesion, antibiofilm activity, immune modulation, and barrier reinforcement as cross-cutting phenomena, it is equally instructive to consider the anti-infective portfolio of each major HMO individually. The structural identity of a particular oligosaccharide determines its receptor-binding specificity, its susceptibility to bacterial glycoside hydrolases, and its capacity to engage immune-associated lectins. The following subsections provide a concise profile of the anti-infective activities documented for each of the principal HMO species, accompanied by summary tables that collate the available experimental and clinical evidence.

### 4.1. Antiviral Activity

#### 4.1.1. 2′-Fucosyllactose (2′-FL)

2′-Fucosyllactose is the most abundant individual HMO in secretor-positive women, constituting up to 30% of total HMOs, and is consequently the most extensively studied species in relation to anti-infective activity. Its structure closely mimics the H(O) blood group antigen expressed on mucosal epithelia, a molecular feature that underpins its broad-spectrum decoy receptor function.

The antiviral repertoire of 2′-FL spans multiple pathogen families. Against respiratory syncytial virus (RSV), 2′-FL acts as a soluble decoy receptor, reducing viral load in 16HBE airway epithelial cells [20]. In an influenza-specific murine vaccination model, dietary 2′-FL improved both humoral and cellular immune responses, an effect partially attributed to direct modulation of immune cell differentiation [10]. Against rotavirus, Laucirica et al. demonstrated that 2′-FL at 5 mg/mL caused a 62% reduction in G1P serotype infectivity when administered post-infection in MA104 cells; notably, rather than purely prophylactic activity, this therapeutic effect distinguishes 2′-FL from many conventional anti-adhesion strategies [21]. 2′-FL also competes with HIV-1 gp120 for binding to DC-SIGN on dendritic cells, potentially limiting viral trans-infection of CD4+ T cells [22]. More recently, Patil et al. demonstrated inhibition of human norovirus GII.4 Sydney replication in human intestinal enteroids derived from both adult and paediatric donors [23]. Lou et al. reported that 2′-FL blocks Coxsackievirus A9 attachment and internalisation with up to 99.97% inhibition at 10 mg/mL, interacting with αvβ6 and FCGRT receptors [24].

Immunologically, 2′-FL attenuates TLR4/NF-κB signalling via miR-146a upregulation, inhibits TLR5 and TLR7 activation [25,26], and binds DC-SIGN to prevent RSV-mediated inhibition of dendritic cell activation [27]. The clinical relevance of these effects was demonstrated by Goehring et al., who found 29–83% lower plasma concentrations of pro-inflammatory cytokines in 2′-FL-supplemented formula-fed infants compared to unsupplemented controls [28]. The antiviral activities of 2′-FL—together with those of the other individual HMOs discussed below—are summarised in Table 1.

#### 4.1.2. 3-Fucosyllactose (3-FL)

The antiviral spectrum of 3-FL is notably broad. Against RSV, 3-FL binds glycoprotein G and reduces viral load in airway epithelial cells [20]. In a murine influenza model, dietary 3-FL enhanced antiviral responses and increased survival rates [32]. A particularly novel finding is the capacity of 3-FL to inhibit SARS-CoV-2 infection; Yu et al. demonstrated competitive binding to the receptor-binding domain (RBD) of the spike protein, inhibiting both direct and trans-binding across three SARS-CoV-2 mutant pseudoviruses, although with weaker potency than 2′-FL [33]. 3-FL also competes with HIV-1 gp120 for DC-SIGN binding via structural mimicry of Lewis antigens (Leᵃ/Leˣ), with DC-SIGN showing high reactivity to its fucose residue [21,22].

Immunologically, 3-FL activates TLR2 while simultaneously inhibiting TLR5, TLR7, and TLR8, producing a net silencing of Th2 effector responses alongside enhancement of IL-10, IL-17, and decreased IL-12p70, IL-13, and IL-23 [39]. Boll et al. further demonstrated that 3-FL exhibits potent barrier enhancement properties, increasing glycocalyx components including hyaluronic acid and heparan sulfate in Caco-2 cells [39].

#### 4.1.3. 3′-Sialyllactose (3′-SL)

3′-Sialyllactose is the prototypical acidic HMO, carrying a sialic acid (Neu5Ac) residue α2,3-linked to the galactose of lactose. This linkage mimics the receptor structures preferentially recognised by avian influenza viruses, endowing 3′-SL with a distinctive antiviral profile that complements the fucosylated species.

The antiviral activity of 3′-SL is particularly well-characterised against influenza. Guo et al. demonstrated that 3′-SL binds the hemagglutinin (HA) protein of influenza H1N1 via its sialylated galactose substructure, achieving an IC_50_ of 33.46 μM in HEP-2 cells and reducing the cytopathic effect and inflammatory storm (TNF-α, IL-6, iNOS), with synergistic effects when combined with osteopontin [34]. Günther et al. showed that 3′-SL-conjugated dendritic polymers (3′-SL-PAMAM dendrimers) inhibited more than 50% of 13 avian influenza virus subtypes including H1N1, H1N2, H3N2, and highly pathogenic H5N1 (MIC = 5 mM) and achieved complete elimination of H9N2 in SPF chickens within 24 h via a colonic wash-out mechanism [35]. Against RSV, 3′-SL reduces viral load as a soluble decoy receptor [20]. As a decoy receptor mimicking intestinal histo-blood group antigens (HBGAs), 3′-SL also inhibits rotavirus VP8* binding to host cells, with enhanced efficacy when combined with 6′-SL [31].

#### 4.1.4. 6′-Sialyllactose (6′-SL)

6′-Sialyllactose carries a sialic acid residue α2,6-linked to galactose the human-type influenza receptor linkage conferring preferential activity against human-adapted influenza strains, in contrast to the avian-tropic 3′-SL.

Günther et al. demonstrated that 6′-SL-PAMAM dendrimers potently inhibit human seasonal influenza strains (H1N1, H3N2) at low millimolar concentrations, with the octavalent (6′-SL)-PAMAM construct being the most potent formulation [22]. However, activity against avian subtypes was limited; Pandey et al. found no significant inhibition of H5N1, H5N8, or H9N2 at concentrations up to 200 mM. [36]. This complementary specificity 3′-SL for avian strains, 6′-SL for human strains reflects the α2,3 versus α2,6 sialic acid linkage preference of avian versus human influenza hemagglutinins. 6′-SL also reduces influenza A viral load in 16HBE airway cells and inhibits rotavirus VP8* binding with enhanced efficacy when combined with 3′-SL [20,31].

#### 4.1.5. Lacto-N-neotetraose (LNnT)

LNnT is the most abundant neutral non-fucosylated tetrasaccharide in human milk (Galβ1,4-GlcNAcβ1,3-Galβ1,4-Glc). Its type 2 chain structure featuring a terminal Galβ1,4-GlcNAc disaccharide serves as a precursor of the H-type II histo-blood group antigen, a molecular feature that underpins its specific receptor interactions.

Against influenza A, LNnT reduces viral load in airway epithelial cells [20]. Against rotavirus, multiple studies document anti-adhesive activity. Donovan and Comstock demonstrated that LNnT acts as a decoy receptor, reduces rotavirus infectivity, and decreases the duration of diarrhoea in an *in vivo* piglet ileal loop model [37]. Most recently, Yan et al. provided a mechanistic refinement, showing that the VP8* protein of porcine rotavirus P [13] specifically binds the LNnT tetrasaccharide (Galβ1,4-GlcNAcβ1,3-Galβ1,4-Glc) and that infection is inhibited by blocking GM1a ganglioside establishing a link between LNnT structure and ganglioside-mediated cell entry [38].

#### 4.1.6. Lacto-N-tetraose (LNT)

LNT is the type 1 chain counterpart of LNnT, featuring a terminal Galβ1,3-GlcNAc disaccharide. This subtle structural difference confers distinct biological properties, particularly in the context of neonatal rotavirus infection and direct antimicrobial activity against Group B Streptococcus.

Ramani et al. reported an unexpected finding in a neonatal cohort study from India: higher LNT concentrations in breast milk were associated with gastrointestinal symptoms in rotavirus-positive neonates infected with the G10P [13] serotype [40].

Critically, LNT does not appear to act as a decoy receptor for this neonatal serovar a finding that underscores the importance of serotype-specific interactions and cautions against extrapolating anti-adhesive effects across all rotavirus strains.

In direct antimicrobial assays, LNT inhibits growth of Group B Streptococcus and acts synergistically with vancomycin and ciprofloxacin [12]. Ruiz-Palacios et al. also documented that LNT inhibits adhesion of *C. jejuni* to intestinal epithelial cells [10].

#### 4.1.7. Lacto-N-triose II (LNT II)

Lacto-N-triose II (GlcNAcβ1,3-Galβ1,4-Glc) is a trisaccharide that serves as the structural core and biosynthetic precursor of both LNT and LNnT.

Li et al. characterised LNT II as the central structural unit from which complex branched HMOs are biosynthesised, suggesting that its biological significance extends beyond direct bioactivity to encompass its role as a metabolic hub in HMO biochemistry [41].

LNTII confers anti-adhesive and antiviral properties.

Although less abundant as a free oligosaccharide in mature milk, LNT II has recently attracted attention for its own distinct bioactivities.

Liu et al. documented direct antimicrobial activity of LNT II, establishing that this trisaccharide inhibits bacterial growth independently of its role as an HMO precursor [42].

### 4.2. Shaping the Intestinal Microbiome: Prebiotic Selectivity and Colonisation Resistance

Perhaps the most extensively documented anti-infective function of HMOs is their selective promotion of beneficial microbiota in the infant gut. Because they resist digestion by host enzymes, HMOs reach the large intestine largely intact, where they serve as preferred carbon sources for specific bacterial groups. The primary consumers are bifidobacteria, particularly *Bifidobacterium longum* subsp. *infantis* (*B. infantis*), which possesses a dedicated 43 kb genomic cluster for HMO catabolism and can use these molecules as its near-sole carbon source [43,44]. This intracellular degradation strategy, mediated by ABC transporters and a suite of glycoside hydrolases (GH95, GH33, GH20, GH2, GH42), contrasts with the extracellular approach of *B. bifidum*, which releases HMO fragments that sustain cross-feeding networks [45,46].

A 2024 randomised clinical trial comparing infants fed a 2′-FL-supplemented formula, another one with galacto-oligosaccharides and fructo-oligosaccharides (GOS/FOS) and exclusively breastfed controls found that the relative abundance of *Bifidobacterium* in the HMO group was statistically comparable to that of breastfed infants [45]. This bifidogenic convergence is clinically meaningful: a *Bifidobacterium*-rich microbiome generates lactate and acetate, which lower intestinal pH and create conditions hostile to pathogens, while cross-feeding of HMO breakdown products supports a broader community of commensal bacteria [47,48]. Wong et al. reviewed the clinical evidence for *B. infantis* strain M-63, which consumed 78.8% of total available HMOs within 10 h *in vitro*, and demonstrated in a double-blind RCT of 110 healthy term infants that supplementation increased *Bifidobacterium* abundance, decreased stool pH, elevated faecal acetic acid, and increased faecal IgA levels [45].

The functional consequences of this microbiome modulation extend beyond the gut. Bajic et al. demonstrated that HMOs significantly increased short-chain fatty acid (SCFA) production acetate, propionate, and butyrate from predicted daily doses as low as 0.3–0.5 g in children and adults, with each HMO exhibiting a distinct SCFA profile: 6′-SL most strongly promoted propionate, LNnT increased butyrate, and 2′-FL and 3′-SL increased acetate [49]. Beyond SCFAs, untargeted metabolomics revealed enhanced production of immune-related metabolites including indole-3-lactic acid and 3-phenyllactic acid, as well as gut–brain axis metabolites such as GABA and acetylcholine [49].

As a prebiotic, LNnT is a preferred substrate for *B. infantis* and most strongly increases butyrate production among the HMOs tested, at doses as low as 0.5 g/day. In the context of antibiotic-induced dysbiosis, Pang et al. demonstrated that LNnT was superior to 2′-FL, 3′-SL, and their mixture for gut microbiota recovery, chiefly promoting *Lactobacillus* while increasing *Bifidobacterium* and decreasing pathogenic *Klebsiella* [50]. LNnT also dose-dependently induces maturation of the gut epithelium in HT-29, Caco-2, and HIEC cells [51].

A clinical trial in 32 healthy adults by Jacobs et al. demonstrated dose-dependent *Bifidobacterium* expansion using HMO-Concentrate from pooled donor breast milk, with shotgun metagenomics revealing decreased levels of genes involved in biofilm formation, bacterial invasion of epithelial cells, and antibiotic resistance, coupled with increased antibiotic biosynthesis pathway levels [52]. These functional shifts persisted through to day 28, well beyond HMO cessation. Critically, the complex mixture of over 200 HMOs in the concentrate could not be recapitulated by individual HMOs or defined mixtures of the 10 most abundant structures low-abundance HMOs appear necessary for maximum effect [52].

### 4.3. Anti-Adhesion Properties: HMOs as Soluble Decoy Receptors

Long before the molecular mechanisms were understood, it was recognised that breastfed infants had lower rates of gastrointestinal and respiratory infections. HMOs are now understood to contribute to this protection through competitive inhibition; because many pathogens bind to carbohydrate structures on the intestinal epithelium before colonising, HMOs, whose structures mimic epithelial glycan motifs, act as soluble decoy receptors, intercepting pathogens before they reach their targets [2,8]. This mechanism has been demonstrated for a wide range of organisms.

For *Campylobacter jejuni*, a leading cause of bacterial diarrhoea in infants, 2′-FL reduced adhesion by 26% and 3-FL by 18%, acting through mimicry of the H(O) blood group antigen. The landmark Morrow et al. prospective study showed higher 2-linked fucosyloligosaccharides in maternal milk were associated with reduced moderate-to-severe diarrhoea [11].

For norovirus, crystal structures show 2′-FL and 3-FL binding at the GII.10 histo-blood group antigen pocket, with high-molecular-weight HMOs showing higher affinity for GII.4 norovirus than monovalent HMOs due to multivalent α-fucose avidity [8]. For rotavirus, the VP8* domain of capsid protein VP4 binds HMOs in strain-specific patterns, with 2′-FL, 3′-SL, 6′-SL, and GOS substantially reducing infectivity of human rotavirus strains G1P [8] and G2P [4,8].

Respiratory pathogens are also susceptible. Duska-McEwen et al. demonstrated that 2′-FL and 3′-SL downregulated the viral load of respiratory syncytial virus (RSV) in 16HBE airway epithelial cells, while LNnT and 6′-SL downregulated influenza A viral load [20]. Rijks et al. reviewed the molecular mechanisms by which HMOs may protect against severe respiratory viral infections and subsequent asthma development, noting that fucosylated HMOs most closely resemble epithelial glycan receptors [27]. Against *Pseudomonas aeruginosa*, the lectin PA-IIL is blocked by milk oligosaccharides containing Lewis a trisaccharide, and 2′-FL reduced *P. aeruginosa* adhesion to Caco-2 cells by 17% while 3-FL achieved 26% reduction [8,53]. For uropathogenic *Escherichia coli* (UPEC), HMOs at 15 mg/mL significantly reduced bacterial internalisation into bladder epithelial cells by blocking MAPK and NF-κB activation; notably, HMOs are present in the urine of breastfed but not common formula-fed neonates [54]. A summary of the anti-adhesive activities of individual HMO structures is provided in Table 2.

### 4.4. Antimicrobial and Antibiofilm Activity: A Rapidly Expanding Frontier

Biofilms are surface-attached microbial communities encased in a self-produced extracellular polymeric substance (EPS) matrix that confers resistance to host immune defences and antibiotic treatment. In the neonatal setting, biofilm-forming pathogens are responsible for device-associated infections, ventilator-associated pneumonia, and persistent mucosal colonisation. The demonstration that HMOs possess direct antibiofilm activity represents one of the most important recent advances in HMOs biology [9,57].

Unlike conventional antimicrobials, whose bactericidal pressure inexorably selects for resistant clones, HMOs appear to act through a fundamentally different paradigm, one that targets the architectural and communicational scaffolding of the biofilm rather than bacterial viability per se. As synthesised by Bhowmik and colleagues, HMOs interfere with several of the sequential steps required for biofilm maturation: initial bacterial adhesion to epithelial surfaces, intercellular signalling via quorum-sensing circuits, and the synthesis and cohesion of the EPS matrix itself [9]. Individual fractions most notably 2′-fucosyllactose (2′-FL), 3′-sialyllactose (3′-SL) and lacto-N-neotetraose have demonstrated activity against a clinically relevant spectrum of neonatal pathogens, including *Streptococcus agalactiae*, *Staphylococcus aureus*, *Acinetobacter baumannii*, *Pseudomonas aeruginosa* and uropathogenic *Escherichia coli* [9].

3′-SL demonstrates direct antimicrobial activity: Kim et al. showed that 3′-SL enhances receptor-mediated endocytosis and phagocytosis of *P. aeruginosa* by THP-1 macrophages, accelerates TLR4/TRIF internalisation, increases ROS generation via Rac1 recruitment, and achieves significant bacterial clearance in bronchoalveolar lavage fluid in BALB/c mice [58]. Against *H. pylori*, 3′-SL competes with the sialic acid-binding adhesin SabA for epithelial binding sites [8]. Sato et al. demonstrated that 3′-SL inhibits *S. mutans* biofilm formation at 100 mM more effectively than xylitol through competitive masking of Neu5Ac glycan-binding sites [59].

It is noteworthy the observation that HMOs can sensitise pre-formed biofilms to conventional antibiotics, restoring susceptibility at concentrations otherwise insufficient for eradication a synergistic profile that, combined with the absence of overt selective pressure, positions this class of glycans as promising adjuvants in an era of escalating antimicrobial resistance [9].

#### 4.4.1. Group B Streptococcus: The Best-Characterised System

The foundational work by Ackerman et al. demonstrated that pooled HMOs at physiological breast milk concentrations (~5 mg/mL) achieved up to 89% growth inhibition and 90% biofilm reduction against *Streptococcus agalactiae* (Group B Streptococcus, GBS) strain CNCTC 10/84 [60]. Crystal violet assays, scanning electron microscopy, and confocal laser scanning microscopy confirmed disrupted biofilm architecture and loss of nutrient channels. A subsequent study expanded testing to 14 donor pools against three GBS strains plus methicillin-resistant *Staphylococcus aureus* (MRSA) USA300. A subsequent study expanded testing against three GBS strains, MRSA USA300, and *A. baumannii* ATCC 19606 [60].

Moore et al. tested pooled HMOs against 30 diverse clinical GBS strains spanning multiple capsular serotypes and sequence types. At 2.5 mg/mL, biofilm inhibition reached 50% in colonising strains and 45% in invasive strains (*p* < 0.0001). Susceptible capsular types included CpsIb, CpsII, CpsIII, CpsV, and CpsVI, while CpsIa, ST-7, and ST-17 were resistant, underscoring important strain-specific heterogeneity [61]. Both biofilm inhibition and biofilm dismantling were demonstrated.

Craft et al. systematically interrogated fucosylated and nonfucosylated HMOs against multiple GBS strains and found that fucose presence alone does not predict antimicrobial or antibiofilm activity; rather, the location and degree of fucosylation are key determinants [62].

Native 2′-FL was devoid of substantial anti-biofilm activity against GBS, but its chemical derivative 1-amino-2′-fucosyllactose reduced biofilm production by 46% (GB2) and 37% (GB590), suggesting that semi-synthetic HMOs derivatives could enhance therapeutic potency [63].

The proposed mechanism involves cationic 1-amino-2′-FL interacting with the anionic EPS matrix and negatively charged extracellular DNA. Sialylated LNT variants were also evaluated, with disialyllacto-N-tetraose (DSLNT) demonstrating antimicrobial activity via increased cellular permeability [64].

#### 4.4.2. Acinetobacter Baumannii: An Emerging Neonatal Threat

*A. baumannii* is an increasingly prevalent nosocomial pathogen in neonatal intensive care units, with multidrug- and pan-drug-resistant strains posing therapeutic challenges. The first evidence of HMO activity against this organism came from Ackerman et al., who demonstrated 30–60% biofilm reduction against *A. baumannii* ATCC 19606 using pooled HMOs from 14 donors [60]. This was subsequently extended by Jarzynka et al., who included *A. baumannii* among seven pathogen species susceptible to mature biofilm eradication [65]. The most comprehensive characterisation was provided by Ackerman et al., who tested pooled HMOs against 18 clinical isolates including multidrug-resistant and pan-drug-resistant strains and reported an 8-fold decrease in biofilm formation, with 14 of 18 isolates (78%) susceptible [60]. These findings are particularly significant given the near-absence of effective therapeutic options against biofilm-forming *A. baumannii* in the neonatal setting.

#### 4.4.3. Eradication of Mature Biofilms

A pivotal advance was reported by Jarzynka et al., who demonstrated that pooled HMOs from nine donors could eradicate mature 48-h biofilms of multiple pathogen species not merely prevent biofilm formation [65]. Seven species were tested, including *Staphylococcus aureus*, *Enterococcus faecalis*, *Enterococcus faecium*, *Klebsiella pneumoniae*, *Acinetobacter baumannii*, *Pseudomonas aeruginosa*, and *Burkholderia cenocepacia* (including clinical isolates). Gram-positive species were predominantly susceptible, with *E. faecalis* being the most sensitive. A critical concentration-dependent finding emerged: at concentrations exceeding 20 mg/mL, bactericidal effects were abolished, likely because oligosaccharides became nutrient sources. Size-exclusion chromatography revealed the antibiofilm active compound was not fucosyllactose; rather, a smaller, non-fucosylated HMO component appeared responsible [65].

Two complementary, non-mutually exclusive mechanisms have been proposed to account for the paradoxical loss of bactericidal activity at supra-physiological concentrations [9,65]. First, when the exogenous oligosaccharide pool exceeds the threshold required for receptor occupancy at the bacterial cell surface, HMOs are progressively redirected from inhibitory ligands toward fermentable carbon substrates, sustaining rather than suppressing bacterial proliferation; this metabolic saturation effect is consistent with the broader observation that excess exogenous glycans abrogate the nutritional advantage that biofilm formation otherwise confers under nutrient-limited conditions [9]. Second, the high local concentration of free oligosaccharides is thought to interfere with the multivalent receptor engagement required for membrane permeabilisation, EPS disruption, and quorum-sensing inhibition, dispersing the active species across an excess of unbound carbohydrate and effectively diluting the inhibitory contact at the bacterial surface [65]. Taken together, these observations indicate that the antibiofilm activity of HMOs follows a non-monotonic, biphasic dose–response profile: at sub-threshold concentrations (below approximately 2–5 mg/mL), receptor occupancy and EPS perturbation are insufficient to disrupt biofilm initiation and quorum-sensing circuits, and the bactericidal effect is correspondingly weak; at intermediate concentrations (5–20 mg/mL), which align closely with the physiological range of mature human milk [2,13], antibiofilm and bactericidal activities are maximal; whereas at supra-physiological concentrations (>20 mg/mL), the protective effect is reversed as HMOs are converted into bacterial substrates and the multivalent inhibitory contacts are diluted [9,65]. Beyond reconciling apparently contradictory *in vitro* observations across studies, this biphasic profile carries direct translational implications for the rational dosing of HMO-supplemented infant formulas and for prospective HMO-based therapeutic preparations, where exposure must be maintained within the physiological window in order to retain anti-infective efficacy.

#### 4.4.4. Antibiofilm Effects Against Other Pathogens

Komatsu et al. provided the first comprehensive review of HMO effects organised by gastrointestinal tract region, documenting anti-adhesion and antibiofilm activity against *Streptococcus mutans* and *Candida albicans* in the oral cavity, alongside decoy receptor activity for *Helicobacter pylori* in the stomach [66]. Sato et al. reported the first RNA-Seq transcriptomic analysis of HMOs effects on oral biofilm gene expression, finding that 6′-SL and N-acetylneuraminic acid significantly inhibited *S. mutans* biofilm formation more effectively than xylitol, while 2′-FL showed no significant inhibitory effect [59]. For *Clostridioides difficile*, both 3′-SL and 6′-SL decreased adhesion to colon cells and reduced biofilm formation [55].

#### 4.4.5. Mechanistic Basis of Antibiofilm Activity

The mechanistic basis of HMOs antibiofilm activity is multifaceted: at least six distinct pathways have been identified, operating at different stages of biofilm development and against different structural targets, as detailed in Table 3.

A comprehensive summary of antimicrobial and antibiofilm activities across individual and pooled HMOs structures is provided in Table 4.

#### 4.4.6. HMO–Antibiotic Synergy: Potentiating Existing Therapeutics

A striking finding is that HMOs potentiate the activity of multiple antibiotic classes against key pathogens. Craft et al. demonstrated that HMOs sensitise GBS to aminoglycosides, macrolides, lincosamides, and tetracyclines on a strain-specific basis, but do not potentiate β-lactams or glycopeptides [71]. The mechanistic basis is that HMOs increase membrane permeability, facilitating entry of intracellularly targeting antibiotics but not those targeting extracellular cell wall synthesis.

Most remarkably, Chambers et al. showed that HMOs sensitise GBS to trimethoprim to which GBS is intrinsically resistant achieving MIC reductions of up to 512-fold across diverse isolates [68]. Global untargeted metabolomics revealed HMOs exposure causes significant perturbations in linoleic acid, sphingolipid, glycerophospholipid, and pyrimidine metabolism.

These findings position HMOs as potential adjuvants to antibiotic therapy in neonatal infections, an application of particular importance given the increasing prevalence of antimicrobial resistance in neonatal pathogens.

### 4.5. Immune Modulation: Direct and Indirect Pathways

During the neonatal period and early life, the adaptive immune system remains functionally immature; protection relies heavily on passive transfer of maternal immunity and on the bioactive components of breast milk. HMOs contribute to this protection through both direct and indirect mechanisms. Directly, they interact with glycan-binding proteins (lectins) on immune and epithelial cells, including galectins, sialic acid-binding immunoglobulin-like lectins (siglecs), selectins, and C-type lectins [26,72]. Indirectly, by fuelling the growth of bifidobacteria and the production of SCFAs, particularly butyrate, they support the maturation of intestinal immune architecture and the epithelial barrier [51].

Immunologically, 3′-SL binds siglec-1, -3, -5, -7, -9, and -10, and inhibits leukocyte adhesion to endothelial cells through selectin interaction [27]. Pessentheiner et al. reported that 3′-SL inhibits TLR4-induced low-grade inflammation in macrophages, attenuates inflammatory gene expression, promotes LXR/SREBP1 activity, reduces histone H3K27 acetylation at LPS-inducible enhancers, and reduces atherosclerosis in a murine model [73].

Boll et al. published a landmark structure–function study testing six HMOs on dendritic cells, macrophages, and T cells. Sialylated HMOs (3′-SL, 6′-SL) promoted secretion of IL-10, IL-12p70, and IL-23 from LPS-activated dendritic cells and M1 macrophages, while increasing IFN-γ and IL-17A from CD4+ T cells, a net Th1/Th17-promoting, Th2-suppressing profile that may help correct neonatal Th2 skewing [50]. Fucosylated HMOs (2′-FL, 3-FL) had minimal immunomodulatory effects on antigen-presenting cells but displayed strong barrier enhancement properties [39]. 2′-FL attenuates TLR4/NF-κB signalling by modulating CD14 expression and increasing miR-146a expression, while LNT II activates all TLRs dose-dependently [25,26]. Immunologically, LNT II displays a remarkable breadth of TLR engagement; Cheng et al. demonstrated dose-dependent activation of all TLRs (TLR2–9) with NF-κB-dependent secretion of both IL-10 and TNFα, a dual pro- and anti-inflammatory profile that distinguishes LNT II from all other individual HMOs tested to date [25].

Treatment of human monocyte-derived dendritic cells with HMO mixtures from pooled human milk increased secretion of the tolerogenic cytokines IL-10 and IL-27, while lowering TNFα and IL-6, and induced differentiation of naïve T cells into T regulatory cells [74].

Rijks et al. provided an extensive analysis of HMOs interactions with the immune system during viral infections. Sialylated HMOs (3′-SL and 6′-SL) were shown to bind Siglec-1, -5, -7, -9, and -10, and to inhibit leukocyte adhesion to endothelial cells via selectin-mediated interactions. Additionally, 2′-FL binds to DC-SIGN, thereby preventing the interaction of other ligands and potentially counteracting RSV-induced inhibition of dendritic cell activation [25,27].

In combination with 2′-FL, 6′-SL reduced ILC2-mediated allergic airway inflammation through increased SCFAs levels in a murine model [75].

SCFAs derived from HMOs fermentation further modulate immune function through G protein-coupled receptors GPR43 and GPR41, with acetate protecting against RSV infection in murine models via activation of GPR43 and increased interferon-stimulated gene expression [27,76].

In an additional analysis from the Goehring et al. randomised controlled trial discussed earlier (Section 4.1), peripheral blood mononuclear cells from formula-fed infants without HMOs secreted significantly higher levels of pro-inflammatory cytokines after ex vivo stimulation with RSV than those from breastfed or 2′-FL-supplemented infants [28].

A structured overview of the immune-modulatory activities of individual and pooled HMOs, organised by receptor interaction and cytokine profile, is provided in Table 5.

### 4.6. Epithelial Barrier Reinforcement

Structure-dependent barrier effects identified by Boll et al. demonstrate that fucosylated HMOs (particularly 3-FL) and neutral non-fucosylated HMOs (LNT, LNT II) display the strongest barrier enhancement, increasing transepithelial electrical resistance in Caco-2 monolayers at physiologically relevant concentrations in both basal and inflammatory conditions [39]. HMOs increase expression of ZO-1, claudin-5, claudin-8, and occludin; promote MUC2 expression in goblet cells through NLRP6 signalling; and induce endoplasmic reticulum chaperones for proper MUC2 folding. 3-FL increases glycocalyx components (hyaluronic acid and heparan sulfate) in Caco-2 cells.

Regarding the lung epithelial barrier, Rijks et al. noted that both RSV and rhinovirus disrupt epithelial barrier integrity through apoptotic cell death and tight junction degradation, and that HMOs could have protective effects on the lung epithelial barrier through their systemic availability [27]. Low concentrations of HMOs have been detected in infant blood and urine, and HMOs are present in amniotic fluid, suggesting potential direct effects on the foetal lung [28]. Butyrate and propionate from HMO fermentation increased tight junction protein expression *in vitro* lung epithelial cell models, suggesting that the gut–lung axis may be a pathway through which HMOs exert distant barrier-protective effects [77].

Zhang et al. comprehensively reviewed how HMOs modulate the intestinal epithelial barrier through three mechanisms: selective prebiotic stimulation of commensal bacteria, provision of soluble decoy receptors that prevent pathogen adhesion, and direct modulation of epithelial cell proliferation, differentiation, and tight junction assembly [46]. The systemic detection of HMOs and their metabolites supports the hypothesis that barrier reinforcement extends beyond the gut to include the respiratory mucosa.

Clinical and preclinical evidence for HMO-mediated barrier reinforcement, prebiotic activity, and infection-related outcomes is summarised in Table 6.

## 5. HMOs and Recovery from Antibiotic-Induced Dysbiosis

Antibiotic exposure in early life is increasingly recognised as a risk factor for subsequent infections and immune dysregulation. HMOs may mitigate the consequences of antibiotic-induced dysbiosis. Pang et al. directly compared LNnT, 3′-SL, 2′-FL, and their mixture for promoting gut microbiota recovery after antibiotic-induced dysbiosis in a mouse model, demonstrating that LNnT was superior to all other tested structures, chiefly promoting recovery of *Lactobacillus* while increasing *Bifidobacterium* levels and decreasing pathogenic *Klebsiella* [50]. In a *C. difficile* infection model secondary to antibiotic therapy within the same study, LNnT attenuated intestinal epithelial damage and decreased inflammatory status representing the first direct head-to-head comparison of individual HMO structures for dysbiosis recovery efficacy [50].

Button et al. demonstrated in 56 antibiotic-treated healthy adults that a synbiotic combining *B. infantis* with HMO-Concentrate achieved 76% engraftment, with maximum relative abundance reaching 81% of the bacterial population [79]. Engraftment was entirely HMO-dependent and was associated with faster acetate recovery, increased indole-3-lactic acid (anti-inflammatory), and decreased p-cresol sulfate (pro-inflammatory); *in vitro*, the synbiotic additionally decreased viability of *Klebsiella pneumoniae*, *Enterobacter cloacae*, and *E. coli* [79].

A broader conceptual framework was provided by Song et al., who reviewed the bidirectional HMO–microbiome axis and proposed that HMOs function as microbiota-derived epigenetic regulators, translating signals from the maternal gut microbiome to shape infant metabolic programming [82]. The clinical relevance of this microbiome-mediated protection was independently demonstrated by Puccio et al., who’s multicenter RCT showed that infants fed formula supplemented with 2′-FL and LNnT had reduced antibiotic use during the first 12 months of life, with faecal community types at 3 months predicting later antibiotic need [30].

## 6. HMOs in Infant Formula: From Regulation to Clinical Practice

The recognition that HMOs represent the largest compositional gap between human milk and infant formula has driven substantial investment in their biotechnological production. By the early 2010s, fermentation-based processes using engineered *Escherichia coli* or Saccharomyces cerevisiae had made it possible to produce individual HMOs at scale in forms structurally identical to those in human milk [8]. Regulatory approval has followed a progressive trajectory: EFSA issued its first safety opinions on 2′-FL and LNnT in 2015, followed by LNT and the 2′-FL/DFL mixture in 2019, 3-FL and the sialylated species 3′-SL and 6′-SL in 2020–2021, and most recently LNFP I, which in 2023 became the first approved pentasaccharide HMO [63,64]. The US FDA has similarly expanded its GRAS portfolio, with clearances now covering 2′-FL, LNnT, LNT, 3-FL, 3′-SL, 6′-SL, the 2′-FL/DFL mixture, and LNFP I. Permitted use levels have also been progressively revised upward as post-market safety evidence has accumulated: the EU maximum for 2′-FL in infant formula was increased from 1.2 g/L to 3.0 g/L in 2024, approaching concentrations found in human colostrum. In 2017, the first commercial infant formula containing 2′-FL and LNnT was launched in the European Union [83,84].

Some standard and partially hydrolysed protein infant formulas (pHF) contain a blend of five human milk oligosaccharides (HMOs): 2′-fucosyllactose (2′-FL), difucosyllactose (DFL), lacto-N-tetraose (LNT), 6′-sialyllactose (6′-SL), and 3′-sialyllactose (3′-SL). These oligosaccharides are structurally identical to those naturally present in human milk and are often combined with Limosilactobacillus reuteri in partially hydrolysed protein formulas.

Initially, some pHF formulas were supplemented with five HMOs; more recently, six-HMO blends have been introduced. These formulations are designed to act synergistically with *Bifidobacterium lactis* and *Bifidobacterium infantis* and include: 2′-fucosyllactose (2′-FL), difucosyllactose (DFL), 3-fucosyllactose (3-FL), lacto-N-tetraose (LNT), 6′-sialyllactose (6′-SL), and 3′-sialyllactose (3′-SL) [80,81].

## 7. Discussion

Quality of nutrition in infancy is a key determinant of somatic growth, neurodevelopment, and long-term health, with substantial implications for morbidity and the risk of chronic diseases in adulthood. Within this framework, breastfeeding is universally recognised as the gold standard in neonatal and infant nutrition, owing to its complex, integrated, and scientifically validated benefits.

Breast milk constitutes a unique, species-specific biofluid, endowed with biological properties that cannot be fully replicated by breast milk substitutes. Its composition is dynamic, evolving over time and finely tailored to the infant’s nutritional, metabolic, and immunological needs, in relation to the developmental stage and the mother–child interaction [85,86].

An in-depth understanding of human milk composition provides a foundational tool for the nutritional management of newborns and infants, with substantial implications for modulating immune responses and enhancing anti-infective protection. Consequently, the promotion and support of natural feeding represent major priorities within public health strategies to reduce infection burden and improve child health outcomes.

The anti-infective advantages of breastfeeding are mediated by a dynamic, bioactive repertoire including secretory immunoglobulins (notably sIgA), lactoferrin and other antimicrobial proteins, HMOs that shape the gut microbiota and inhibit pathogen adhesion, immune cells, and a range of cytokines and chemokines that act in concert to bolster mucosal and systemic immunity across developmental stages [87].

Human milk oligosaccharides have been the subject of sustained scientific investigation for over a century, owing to their structural complexity, extensive diversity, and multifunctional roles in antimicrobial defence and immune modulation. These bioactive glycans are now recognised as key contributors in early life protection, especially in the neonatal period, mediating both direct and indirect inhibition of pathogens, promoting the establishment of beneficial microbiota, and shaping innate and adaptive immune responses [3].

The anti-infective properties of HMOs operate through several interconnected mechanisms: glycan mimicry-based competitive inhibition of pathogen adhesion, direct antimicrobial and antibiofilm activity against clinically relevant neonatal pathogens, selective prebiotic shaping of the microbiome to establish colonisation resistance, modulation of innate and adaptive immunity, and reinforcement of epithelial barriers in both the gut and lungs.

The antibiofilm data spanning GBS, MRSA, *A. baumannii*, *E. faecalis*, *S. mutans*, and *C. difficile* demonstrate both biofilm prevention and mature biofilm eradication capabilities. The concentration window for optimal activity (5–20 mg/mL) aligns with physiological breast milk concentrations. The antibiotic-potentiating effects of HMOs represent an underexplored therapeutic avenue for neonatal and infant infections. The structure-dependent nature of antibiofilm activity where pooled HMO mixtures consistently outperform individual components supports the concept that the more than 200 distinct oligosaccharide structures present in human milk have evolved as a combinatorial defence system, in which structural diversity is required to achieve full biological efficacy.

Several key questions in this field remain unresolved. In particular, it is not yet clear which combinations of human milk oligosaccharides (HMOs), administered at defined concentrations and ratios, confer maximal anti-infective efficacy. Available evidence indicates that individual HMOs exert distinct, context-dependent effects, whereas combinations may produce synergistic outcomes through complementary mechanisms, including inhibition of pathogen adhesion, modulation of the gut microbiota, reinforcement of epithelial barrier integrity, and regulation of host immune responses. The optimal composition of such mixtures is unlikely to be universal, but instead shaped by host and environment-specific factors, including infant age, gestational maturity, baseline microbiota composition, environmental exposures, and susceptibility to specific pathogens.

Against this backdrop, further well-designed clinical trials and mechanistic studies are required to clarify dose–response relationships, identify the most effective HMO compositions, and determine whether targeted formulations can confer enhanced protection against specific infectious diseases. Addressing these challenges will be critical for the rational design of next-generation infant formulas that more closely recapitulate the protective biological functions of human milk.

## 8. Conclusions and Future Directions

Human milk oligosaccharides occupy a unique position in the landscape of infant nutrition and defence against infection.

Over time, knowledge about HMOs has evolved from an unidentified “bifidus factor” to a class of molecules whose structural diversity, prebiotic selectivity, antibiofilm activity, immunomodulatory effects, and contribution to barrier integrity are now reasonably well characterised.

Human milk oligosaccharides (HMOs) constitute an indispensable component of the early nutritional environment, conferring anti-infective protection with potential effects that extend beyond the period of breastfeeding.

Translating these advances into routine clinical practice will require a coordinated programme of next-generation trials specifically designed to resolve the three principal uncertainties that currently constrain rational HMO use, namely the identification of optimal combinations, the characterisation of dose–response relationships, and the evaluation of efficacy in clinically vulnerable populations.

With respect to combination strategies, future trials should adopt factorial or response-surface designs that compare individual HMOs against rationally selected mixtures rather than against unsupplemented controls alone, allowing a quantitative dissection of additive, synergistic, and antagonistic interactions between structurally distinct species [8,30]. Mechanistically informative pairings warrant prioritisation, including 2′-FL with LNnT for combined anti-adhesive and bifidogenic effects [30,81]; 3′-SL with 6′-SL for complementary coverage of avian-tropic and human-tropic influenza receptors [31,35]; and structurally diverse pooled blends that more closely approximate the natural HMO repertoire of human milk [12,80], in order to test whether the combinatorial complexity observed in vivo is genuinely required for full biological efficacy or whether a defined subset of structures is sufficient.

With respect to the dose–response relationship, formal dose-ranging trials anchored to the physiological window of 5–20 mg/mL identified in antibiofilm studies are needed [9,65], with stratification according to lactation stage to account for the natural decline in HMO concentration from approximately 20–25 g/L in colostrum to 10–15 g/L in mature milk [2,13]. Pharmacokinetic substudies measuring HMO concentrations in plasma, urine, and stool would be particularly valuable for clarifying the relationship between enteral dose, systemic exposure, and microbial fermentation [43,49], and for identifying any threshold beyond which incremental supplementation no longer translates into proportionate clinical benefit. Such data would also inform whether *in vitro* concentration windows can reasonably be extrapolated to enteral dosing in formula-fed infants, given the dilutional and compartmentalisation effects of *in vivo* exposure.

Population-specific trials are particularly needed in groups in whom HMO exposure is reduced or absent, including preterm and very-low-birth-weight infants at increased risk of necrotising enterocolitis [51,81], infants of non-secretor mothers (who lack 2′-FL in their milk) [5,15], formula-fed infants in low- and middle-income settings characterised by a high gastrointestinal infection burden [3,30], and infants undergoing or recovering from antibiotic therapy [50,80]. Trial endpoints should extend beyond growth parameters and gastrointestinal tolerability to include clinically meaningful infection-related outcomes, such as the incidence of gastrointestinal and lower respiratory tract infections, antibiotic prescriptions, hospital admissions, and necrotising enterocolitis [30], supplemented by mechanistic biomarkers including faecal *Bifidobacterium* abundance, short-chain fatty acid profiles, faecal secretory IgA, and circulating inflammatory cytokine panels [28,75,76]. Adequate follow-up duration, ideally extending into the second and third year of life, is essential for capturing delayed effects on atopic and metabolic outcomes that may follow early microbiome programming [27,47]. Adaptive and platform trial designs would further accelerate progress by allowing simultaneous evaluation of multiple HMO formulations, doses, and target populations within a single methodological framework, while preserving statistical efficiency and ethical acceptability.

A deeper mechanistic understanding of HMO-mediated effects, integrating glycomics, metagenomics, and host immunological readouts [12,43,82], will be essential for translating these recommendations into clinically actionable interventions and for harnessing the full preventive potential of human milk oligosaccharides in early life.

What is clear is that HMOs are not an optional supplement to infant nutrition: they are an essential component of the earliest nutritional environment, with multifunctional properties and effects that may extend well beyond the breastfeeding period.

## Figures and Tables

**Table 1:** Antiviral profile of individual human milk oligosaccharides.

HMO	Target	Mechanism/Key Finding	Model	Evidence Type	Reference
***2′-Fucosyllactose (2′-FL)***					
2′-FL	RSV	Soluble decoy receptor; reduced viral load in 16HBE airway epithelial cells	16HBE cell line	*In vitro*	[20,29]
2′-FL	Influenza	Improved humoral and cellular immune responses to vaccination; direct immune cell modulation	Murine vaccination model	*Animal*	[29]
2′-FL	Rotavirus G1P	62% reduction in infectivity post-infection at 5 mg/mL; therapeutic activity	MA104 cells	*In vitro*	[21]
2′-FL	HIV-1	Competes with gp120 for DC-SIGN binding	Raji-DC-SIGN cells	*In vitro*	[22]
2′-FL	Norovirus GII.4 Sydney	Inhibits replication in human intestinal enteroids (adult and paediatric donors)	Human intestinal enteroids	*In vitro*	[23]
2′-FL	Coxsackievirus A9	Blocks attachment (48.4%) and internalisation (51.3%); 99.97% inhibition at 10 mg/mL; interacts with αvβ6 and FCGRT	RD cells	*In vitro*	[24]
2′-FL + LNnT	Respiratory infections	Fewer bronchitis episodes; reduced antibiotic use	Multicentre RCT (n > 160 healthy term infants)	*Infant clinical*	[30]
***3-Fucosyllactose (3-FL)***					
3-FL	RSV	Binds glycoprotein G; reduces viral load in airway epithelial cells	16HBE cell line	*In vitro*	[31]
3-FL	Influenza	Enhanced antiviral response; increased survival rate	Murine model	*Animal*	[32]
3-FL	SARS-CoV-2	Competitive binding to spike-protein RBD; inhibits direct and trans-binding; confirmed on three mutant pseudoviruses; weaker than 2′-FL	Pseudovirus binding/inhibition assay	*In vitro*	[33]
3-FL	HIV-1	Competes with gp120 for DC-SIGN via Lewis-antigen mimicry (Leᵃ/Leˣ)	Cell-based binding assay	*In vitro*	[21,31]
***3′-Sialyllactose (3′-SL)***					
3′-SL	RSV	Soluble decoy receptor; reduced viral load	16HBE cell line	*In vitro*	[20]
3′-SL	Influenza H1N1	Binds HA via sialylated galactose; IC_50_ = 33.46 μM; reduces cytopathic effect and inflammatory storm; synergistic with osteopontin	HEP-2 cells	*In vitro*	[34]
3′-SL (PAMAM-conjugated)	13 avian influenza subtypes	3′-SL-PAMAM dendrimers: MIC ≤15.62 mM for >50% of subtypes; H5N1 MIC = 5 mM; complete H9N2 elimination in vivo within 24 h	MDCK cells + SPF chickens	*Animal*	[35]
3′-SL	Porcine rotavirus	Decoy receptor mimicking HBGA; inhibits VP8* binding; more effective combined with 6′-SL	Cell-based binding assay	*In vitro*	[31]
***6′-Sialyllactose (6′-SL)***					
6′-SL (PAMAM-conjugated)	Human Influenza H1N1, H3N2 (seasonal)	6′-SL-PAMAM dendrimers inhibit human IAV at low mM; (6′-SL)_8_-PAMAM most potent; also inhibits swine H1N1	Erythrocytes, MDCK, A549	*In vitro*	[35]
6′-SL	Influenza A	Reduced viral load in airway epithelial cells	16HBE cell line	*In vitro*	[20]
6′-SL	Avian influenza (H5N1, H5N8, H9N2)	Limited activity; no inhibition at ≤200 mM; weaker than 3′-SL for avian subtypes	MDCK cells, hemagglutination-inhibition assay	*In vitro*	[36]
6′-SL	Porcine rotavirus	Decoy receptor; inhibits VP8* binding; more effective combined with 3′-SL	Cell-based binding assay	*In vitro*	[31]
***Lacto-N-neotetraose (LNnT)***					
LNnT	Influenza A	Reduces viral load in airway epithelial cells	16HBE cell line	*In vitro*	[20]
LNnT	Rotavirus	Decoy receptor; reduces infectivity; decreases diarrhoea duration in vivo	Piglet ileal-loop model	*Animal*	[37]
LNnT	Porcine rotavirus P [13]	VP8* binds LNnT; infection inhibited by blocking GM1a ganglioside	Binding assay + cell infection	*Mechanistic*	[38]

Evidence-type colour key: ■ *In vitro (light blue)*. ■ *Animal experimental (light lavender)*. ■ *Infant clinical (light green)*. ■ *Adult clinical (light cream)*. ■ *Mechanistic/speculative (light pink). Abbreviations: 2′-FL, 2′-fucosyllactose; 3-FL, 3-fucosyllactose; 3′-SL, 3′-sialyllactose; 6′-SL, 6′-sialyllactose; LNnT, lacto-N-neotetraose; HA, hemagglutinin; HBGA, histo-blood group antigen; IAV, influenza A virus; IC_50_, half-maximal inhibitory concentration; MIC, minimum inhibitory concentration; PAMAM, polyamidoamine; RBD, receptor-binding domain; RCT, randomised controlled trial; RSV, respiratory syncytial virus; SARS-CoV-2, severe acute respiratory syndrome coronavirus 2; SPF, specific-pathogen-free*.

**Table 2:** Anti-adhesion activities of human milk oligosaccharides.

HMO	Pathogen	Anti-Adhesion Mechanism/Outcome	Model/Experimental System	Evidence Type	Reference
Pooled HMOs	*Campylobacter jejuni*	Inhibit binding to intestinal H(O) antigen Fucα1,2Galβ1,4GlcNAc	Caco-2 cell line; mouse intestinal model	*Animal*	[10]
2′-FL	*Campylobacter jejuni*	Reduces faecal Campylobacter colonisation in suckling mice expressing α1,2-fucosyltransferase	Transgenic mouse pup	*Animal*	[10]
2′-FL, 3-FL	*Pseudomonas aeruginosa*, enteric pathogens	Inhibit adhesion to intestinal (HT-29, Caco-2) and respiratory (A549) epithelial cell lines	Multi-cell-line panel	*In vitro*	[53]
Pooled HMOs (fucosyl-rich)	Uropathogenic *E. coli* (UPEC)	Reduce invasion and cytotoxicity in bladder epithelial cells	Bladder epithelial cells	*In vitro*	[54]
3′-SL, 6′-SL	*Clostridioides difficile*	Reduce adhesion and biofilm formation on intestinal cells	Intestinal cell adhesion assay	*In vitro*	[55]
6′-SL	*Pseudomonas aeruginosa*	Reduces internalisation in human pneumocyte cell line	A549 pneumocytes	*In vitro*	[56]
Pooled HMOs	Norovirus	Glycan-mimicry inhibition of HBGA-mediated binding	Salivary HBGA binding assay	*Mechanistic*	[57]

Evidence-type colour key: ■ *In vitro (light blue)*. ■ *Animal experimental (light lavender)*. ■ *Infant clinical (light green)*. ■ *Adult clinical (light cream)*. ■ *Mechanistic/speculative (light pink). Abbreviations: 2′-FL, 2′-fucosyllactose; 3-FL, 3-fucosyllactose; 3′-SL, 3′-sialyllactose; 6′-SL, 6′-sialyllactose; HBGA, histo-blood group antigen; UPEC, uropathogenic Escherichia coli*.

**Table 3:** Antibiofilm mechanisms and quantitative findings.

HMO/Structure	Pathogen/Biofilm	Mechanism/Quantitative Finding	Model/System	Evidence Type	Reference
Pooled HMOs	Group B Streptococcus (GBS)	Disruption of EPS matrix; quorum-sensing interference; restoration of antibiotic susceptibility	Static and dynamic biofilm assays	*In vitro*	[61,67]
Sialylated LNT variants	Group B Streptococcus	Antimicrobial activity against GBS strains	Multi-strain GBS panel	*In vitro*	[64]
1-amino-2′-FL	*Streptococcus agalactiae*	Inhibits biofilm formation	*S. agalactiae* biofilm assay	*In vitro*	[63]
Pooled HMOs	Group B Streptococcus + trimethoprim	Untargeted metabolomics: HMO-induced metabolic perturbations restore trimethoprim susceptibility despite antifolate resistance	Metabolomics + susceptibility testing	*Mechanistic*	[68]
Pooled HMOs	Group B Streptococcus vaginal colonisation	Reduce murine vaginal colonisation with minimal vaginal microbiota disruption	Murine vaginal colonisation model	*Animal*	[69]
Pooled HMOs	Multidrug-resistant *Acinetobacter baumannii*	Antibiofilm activity against MDR and susceptible isolates	Clinical *A. baumannii* isolates	*In vitro*	[70]
Pooled HMOs (5–20 mg/mL)	Mature multi-pathogen biofilms	Eradication of mature biofilms; biphasic dose–response (loss of activity > 20 mg/mL—see Section 4.4.3)	Mature biofilm eradication assay	*In vitro*	[65]
6′-SL, N-acetylneuraminic acid	*Streptococcus mutans*	RNA-Seq: downregulation of biofilm-formation pathways	RNA-Seq transcriptomics	*In vitro*	[59]
α2,3-/α2,6-sialyllactose	Multiple bacterial pathogens	Enhanced bacterial clearance via receptor-mediated endocytosis and phagocytosis	Phagocytosis assay	*In vitro*	[58]

*Abbreviations: EPS, extracellular polymeric substance; GBS, Group B Streptococcus; HMO, human milk oligosaccharide; LNT, lacto-N-tetraose; MDR, multidrug-resistant*.

**Table 4:** Consolidated antimicrobial and antibiofilm activities of HMOs.

HMO/Fraction	Target Organism	Activity/Quantitative Outcome	Model/System	Evidence Type	Reference
Pooled HMOs	Group B Streptococcus (*Streptococcus agalactiae*)	Antimicrobial and antibiofilm activity; sensitisation to clindamycin, erythromycin, gentamicin, minocycline (strain-specific)	Clinical GBS strains	*In vitro*	[71]
Pooled HMOs	*Staphylococcus aureus*	Antimicrobial and antibiofilm activity	*S. aureus* biofilm assay	*In vitro*	[60]
Pooled HMOs	*Acinetobacter baumannii*	Antimicrobial and antibiofilm activity	Clinical isolates	*In vitro*	[60]
Fucosylated HMOs	Group B Streptococcus	Interrogation of fucosylation patterns; identification of antimicrobial trends	Structure–activity relationship study	*Mechanistic*	[62]
Pooled HMOs	Group B Streptococcus (diverse capsular/sequence types)	Antibiofilm activity across capsular and sequence types	Multi-strain GBS panel	*In vitro*	[61]
Pooled HMOs (review)	Multiple pathogens	Comprehensive review of HMO antibiofilm potential	Narrative synthesis	*Mechanistic*	[9]

*Abbreviations: GBS, Group B Streptococcus; HMO, human milk oligosaccharide*.

**Table 5:** Immune-modulatory activities of human milk oligosaccharides.

HMO	Target/Pathway	Effect/Quantitative Finding	Model/System	Evidence Type	Reference
LNT2 (acid-hydrolysed HMOs)	TLR signalling	Dose- and structure-dependent immunomodulation via TLRs	HEK-Blue TLR reporter cells	*In vitro*	[25]
2′-FL	TLR4/NF-κB/miR-146a	Attenuates β-lactoglobulin-induced food allergy via miR-146a–TLR4–NF-κB axis	Murine food-allergy model	*Animal*	[26]
Pooled HMOs	Dendritic cells	Promote immune tolerance via direct interaction with human dendritic cells	Human moDCs	*In vitro*	[74]
3′-SL	Macrophage polarisation	Reduces low-grade inflammation; attenuates atherosclerosis development	Murine atherosclerosis model	*Animal*	[73]
2′-FL + 6′-SL	ILC2/SCFA	Reduce ILC2-mediated airway inflammation via increased SCFA levels	Murine allergic airway model	*Animal*	[75]
HMO-derived acetate	GPR43/type-1 IFN	Microbiota-derived acetate protects against RSV via GPR43–type-1 interferon response	Murine RSV-infection model	*Animal*	[76]
Butyrate, propionate (HMO-derived SCFAs)	Bronchial-epithelial barrier	Restore cytokine- and HDM-compromised barrier function	Human bronchial epithelial cells	*In vitro*	[77]
2′-FL	Plasma cytokines + PBMC	29–83% lower IL-1ra, IL-6, IL-1β, TNFα, IFNγ vs. unsupplemented formula; cytokine levels comparable to breastfed; lower ex vivo PBMC response to RSV	RCT in healthy term infants	*Infant clinical*	[28]
Pooled HMOs (review)	Innate + adaptive immunity	Multi-mechanism immunomodulation in early life	Narrative synthesis	*Mechanistic*	[14,72]

*Abbreviations: HDM, house dust mite; ILC2, group 2 innate lymphoid cell; IL, interleukin; IFNγ, interferon-γ; LNT, lacto-N-tetraose; moDCs, monocyte-derived dendritic cells; PBMC, peripheral blood mononuclear cell; RCT, randomised controlled trial; RSV, respiratory syncytial virus; SCFA, short-chain fatty acid; TLR, Toll-like receptor; TNFα, tumour necrosis factor-α*.

**Table 6:** Barrier reinforcement, microbiome modulation, and clinical outcomes.

HMO/Formulation	Target Tissue/Outcome	Effect/Clinical or Mechanistic Finding	Model/Population	Evidence Type	Reference
Pooled HMOs	Gut barrier integrity	Differential support of gut-barrier integrity; enhanced Th1 and Th17 effector responses	Caco-2 + T-cell co-culture	*In vitro*	[39]
HMOs + *Bifidobacterium bifidum*	Adhesion to intestinal epithelium	Structural diversity mediates *B. bifidum*-induced epithelial adhesion	HT-29/Caco-2 co-culture	*In vitro*	[78]
Synbiotic: HMOs + *B. infantis*	Microbiome engraftment (adults)	Reversible engraftment in healthy adult microbiomes without antibiotics	Healthy adult RCT	*Adult clinical*	[79]
Hydrolysed whey + 6 HMOs + *B. infantis* + *B. lactis*	Infant tolerability + microbiome	Safe; well tolerated; favourable shifts in faecal microbiome and SCFA profile	Multicentre infant RCT	*Infant clinical*	[80]
5-HMO formula	Faecal microbiome composition	Shifts faecal microbiome of formula-fed infants closer to that of breastfed infants	Infant cohort	*Infant clinical*	[81]
Pooled HMOs (review)	GI barrier + immunity + neurocognition	Integrative review of HMO supplementation in infant formula	Narrative synthesis	*Mechanistic*	[51]
Pooled HMOs (mixed)	Adult gut microbiome modulation	Modulate the intestinal microbiome of healthy adults	Adult RCT	*Adult clinical*	[52]

*Abbreviations: GI, gastrointestinal; HMO, human milk oligosaccharide; RCT, randomised controlled trial; SCFA, short-chain fatty acid*.

## Data Availability

No new data were created or analysed in this study. Data sharing is not applicable to this article.

## Abbreviations

AAI, allergic airway inflammation; ABC, ATP-binding cassette; CLSM, confocal laser scanning microscopy; CMPA, cow’s milk protein allergy; DC-SIGN, dendritic cell-specific intercellular adhesion molecule-3-grabbing non-integrin; DSLNT, disialyllacto-N-tetraose; eDNA, extracellular DNA; EFSA, European Food Safety Authority; EPS, extracellular polymeric substance; ESPGHAN, European Society for Paediatric Gastroenterology, Hepatology and Nutrition; FDA, Food and Drug Administration; FUT2, fucosyltransferase 2; FUT3, fucosyltransferase 3; Fuc, fucose; Gal, galactose; GBS, Group B Streptococcus; GH, glycoside hydrolase; GlcNAc, N-acetylglucosamine; Glc, glucose; GPR41, G protein-coupled receptor 41; GPR43, G protein-coupled receptor 43; GRAS, Generally Recognised as Safe; HDM, house dust mite; HPLC, high-performance liquid chromatography; HMO, human milk oligosaccharide; IEC, intestinal epithelial cell; IgA, immunoglobulin A; IgE, immunoglobulin E; ILC2, group 2 innate lymphoid cells; LNnT, lacto-N-neotetraose; LNT, lacto-N-tetraose; LNT II, lacto-N-triose II; MDR, multidrug-resistant; MIC, minimum inhibitory concentration; moDC, monocyte-derived dendritic cell; MRSA, methicillin-resistant *Staphylococcus aureus*; NEC, necrotizing enterocolitis; NeuAc, N-acetylneuraminic acid; NF-κB, nuclear factor kappa-light-chain-enhancer of activated B cells; PBMC, peripheral blood mononuclear cell; PDR, pan-drug-resistant; RCT, randomised controlled trial; RSV, respiratory syncytial virus; SCFA, short-chain fatty acids; SEM, scanning electron microscopy; Th1, T helper 1; Th2, T helper 2; Th17, T helper 17; TLR, Toll-like receptor; Treg, regulatory T cell; UPEC, uropathogenic *Escherichia coli*; VLBW, very low birth weight; WHO, World Health Organization; 2′-FL, 2′-fucosyllactose; 3-FL, 3-fucosyllactose; 3′-SL, 3′-sialyllactose; 6′-SL, 6′-sialyllactose; GOS/FOS, galacto-oligosaccharides and fructo-oligosaccharides.
